# Functional Screen of Paracrine Signals in Breast Carcinoma Fibroblasts

**DOI:** 10.1371/journal.pone.0046685

**Published:** 2012-10-08

**Authors:** Gui Su, Kyung E. Sung, David J. Beebe, Andreas Friedl

**Affiliations:** 1 Department of Pathology and Laboratory Medicine, University of Wisconsin-Madison, Madison, Wisconsin, United States of America; 2 Department of Biomedical Engineering, University of Wisconsin-Madison, Madison, Wisconsin, United States of America; 3 Pathology and Laboratory Medicine Service, William S. Middleton Memorial Veterans Hospital, Department of Veterans Affairs Medical Center, Madison, Wisconsin, United States of America; Ghent University, Belgium

## Abstract

Stromal fibroblasts actively participate in normal mammary gland homeostasis and in breast carcinoma growth and progression by secreting paracrine factors; however, little is known about the identity of paracrine mediators in individual patients. The purpose of this study was to characterize paracrine signaling pathways between breast carcinoma cells and breast carcinoma-associated fibroblasts (CAF) or normal mammary fibroblasts (NF), respectively. CAF and NF were isolated from breast carcinoma tissue samples and adjacent normal mammary gland tissue of 28 patients. The fibroblasts were grown in 3D collagen gel co-culture with T47D human breast carcinoma cells and T47D cell growth was measured. CAF stimulated T47D cell growth to a significantly greater degree than NF. We detected a considerable inter-individual heterogeneity of paracrine interactions but identified FGF2, HB-EGF, heparanase-1 and SDF1 as factors that were consistently responsible for the activity of carcinoma-associated fibroblasts. CAF from low-grade but not high-grade carcinomas required insulin-like growth factor 1 and transforming growth factor beta 1 to stimulate carcinoma growth. Paradoxically, blocking of membrane-type 1 matrix metalloprotease stimulated T47D cell growth in co-culture with NF. The results were largely mirrored by treating the fibroblasts with siRNA oligonucleotides prior to co-culture, implicating the fibroblasts as principal production site for the secreted mediators. In summary, we identify a paracrine signaling network with inter-individual commonalities and differences. These findings have significant implications for the design of stroma-targeted therapies.

## Introduction

Tumor development and progression are governed by continuous and reciprocal interactions between tumor cells and their surrounding microenvironment. As carcinomas are initiated and progress, the tumor stroma co-evolves with the carcinoma cells, and creates a tumor permissive microenvironment [1], [2]. Gene expression profiling has identified numerous differences between normal and cancerous stroma in the breast [3], [4], [5], [6] and ample evidence supports the notion that stroma is a key driver of tumor development. For example, a recent study found that mammary stroma acquires expression profiles of tumor stroma before the carcinoma becomes invasive [7]. Carcinoma associated fibroblasts (CAF), a key component in breast cancer stroma, actively participate in tumorigenesis by modifying paracrine stroma-carcinoma signaling and extracellular matrix (ECM) [8]. Candidate gene approaches have identified individual paracrine factors such as stroma-derived factor 1 (SDF-1) and hepatocyte growth factor/scatter factor (HGF/SF) as critical for breast carcinoma growth and progression [9], [10]. However, information about the hierarchy of these factors is currently lacking and it is unknown, how universally the factors are involved in patients. Breast cancer is a highly heterogeneous disease and tumors can be segregated into subclasses according to global gene expression profiles. This diversity is not limited to the epithelium alone but extends to the stromal compartment [6], [11], [12]. In fact, stromal gene expression signatures are a powerful predictor of survival [11], [12].

The aim of this work was to identify paracrine carcinoma growth-promoting pathways using fibroblasts isolated from patient tumors and to characterize the variability of these signals between patients. This was accomplished in microchannel 3D co-culture of primary, patient-derived fibroblasts with T47D breast carcinoma cells, using an inhibitor screen. We selected 11 paracrine factor targets, including growth factors, enzymes, and cytokines with known functions in stroma-carcinoma communications [9], [13], [14], [15], [16], [17], [18], [19], [20], [21], [22], [23], [24], [25], [26].

We found that fibroblast growth factor 2 (FGF-2), heparan sulfate-binding epidermal-like growth factor (HB-EGF), heparanase-1, membrane-type 1 matrix metalloproteinase (MT1-MMP), stroma-derived factor 1 (SDF-1) and transforming growth factor beta 1 (TGF-β1) are required for carcinoma cell growth stimulation by CAF from the majority of patients. Conversely, the inhibition of MT1-MMP stimulated carcinoma cell proliferation in co-culture with normal mammary fibroblasts (NF), highlighting the dual roles of this enzyme in tissue homeostasis and tumorigenesis. These findings expose a striking complexity of the paracrine signaling network with implications for potential stroma-targeted therapy.

## Materials and Methods

### Antibodies and Reagents

Neutralizing antibodies to paracrine mediators were obtained commercially (**Table S1**). Mouse anti-human pan-cytokeratin (CK) and rabbit anti-human vimentin antibodies were purchased from Thermo Fisher Scientific (Fremont, CA), mouse monoclonal anti-human smooth muscle actin antibody from Sigma Aldrich (St. Louis, MO). Polyclonal rabbit antibodies to TGF-β1 and IGF-1 for immunohistochemical labeling were from Santa Cruz Biotechnology (Santa Cruz, CA) and Abcam (Cambridge, MA), respectively. Type I rat tail collagen was from BD Biosciences (Bedford, MA), collagenase I and hyaluronidase from Sigma (St. Louis, MO). On Target Plus Smart Pool siRNA oligonuleotides were purchased from Dharmacon Inc. (Lafayette, CO). Microchannel devices were a gift from BellBrook Labs Inc. (Madison, WI).

### Cell Culture

The human breast carcinoma cell line T47D was obtained from Dr. V. Craig Jordan who had originally purchased the cells from the American Type Culture Collection (ATCC, Manassas, VA). The cells were kept as frozen stocks and maintained in DMEM plus 10% fetal bovine serum. The cells were authenticated as T47D cells by karyotyping in August of 2011. Normal mammary fibroblasts immortalized with human telomerase were provided by Dr. C. Kuperwasser in 2007 [27] and maintained as frozen stocks. The cells are grown in DMEM supplemented with 10% calf serum for no longer than 3 months. Cytogenetic analysis in August of 2011 revealed a human diploid (modal chromosome count 47) karyotype with some polyploid cells. These cells were originally named RMF/EG and are referred to as human mammary fibroblasts (HMF) herein. All cells are regularly tested for mycoplasma.

### Tissue Samples and Primary Mammary Fibroblast Isolation

The study was deemed exempt by the Health Sciences Institutional Review Board of the University of Wisconsin – Madison because all samples were de-identified. A waiver of consent was granted. Therefore, no informed consent was performed. Tissue samples were collected from fresh surgical specimens (mastectomies and excisional biopsies) from 28 patients with invasive breast carcinomas. Approximately 500 mm^3^ each were taken from grossly recognizable tumor and adjacent normal breast tissue (distance from carcinoma edge grossly at least 10 mm). In compliance with the IRB protocol, no patient information was obtained. H&E stained sections from formalin-fixed, paraffin-embedded tissue were prepared from each tissue sample to confirm benignity or malignancy and to obtain information about histological subtype and pathological grade. A tissue microarray (TMA) was prepared from these blocks and used for estrogen receptor (ER) and progesterone receptor (PR) measurement as described [28]. Slides from this TMA were also used for immunohistochemical analysis of TGF-β1 (rabbit polyclonal, 1∶50) and IGF-1 (rabbit polyclonal, 1∶400) following a immunoperoxidase protocol. Primary breast fibroblast culture was established as previously described [26]. Epithelial cell contamination in primary fibroblast cultures was estimated as the percentage of cells displaying positive CK staining. Confluent primary fibroblast cultures contain less than 1% of epithelial cell contamination. The primary cells were grown for no longer than 4 weeks.

### Microchannel Collagen Gel Co-culture

**Figure 1:**
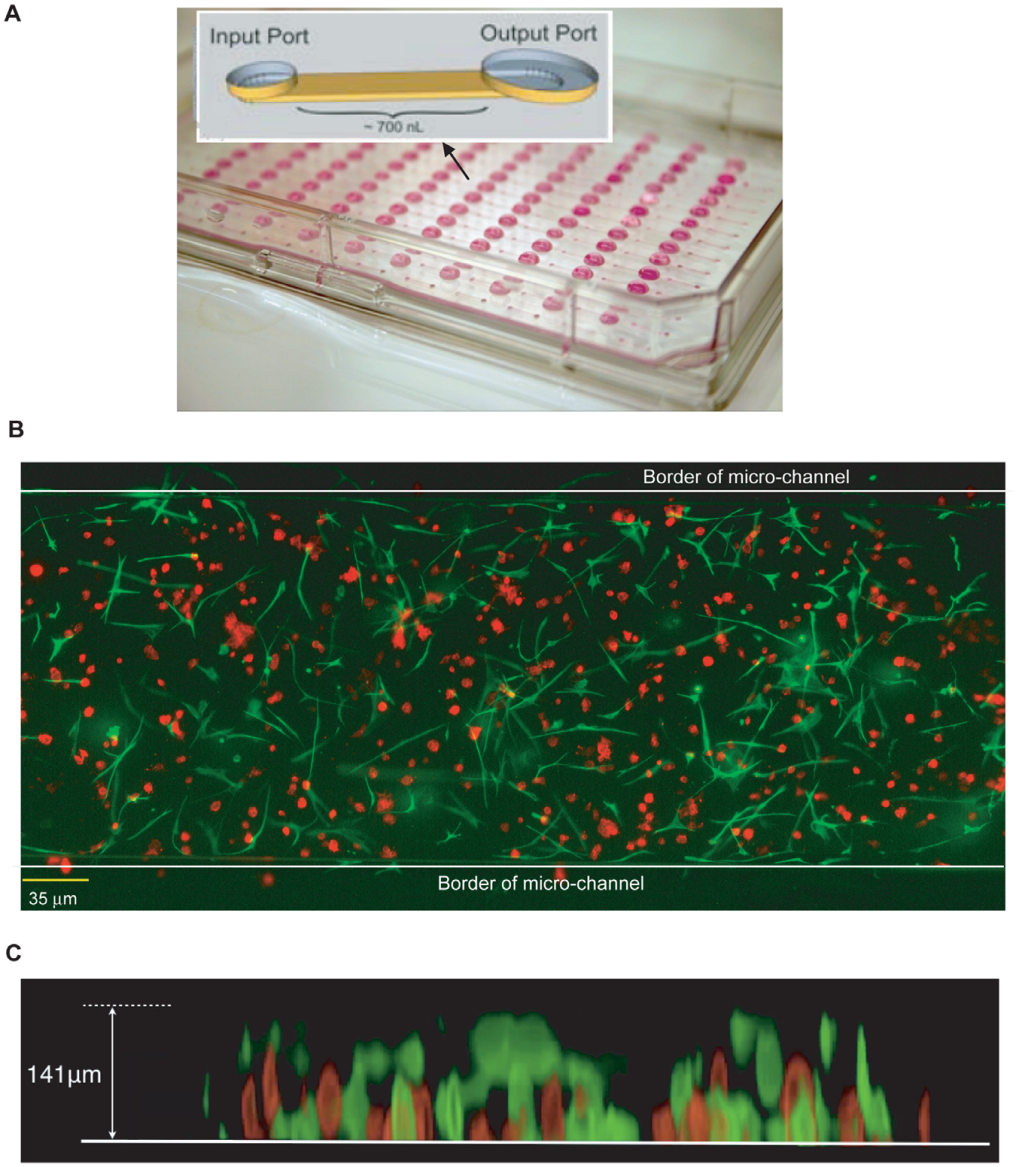
Immunocytochemical staining of array-based micro-channel 3D collagen gel co-culture of T47D cells and HMF. A. Image of micro-channel device. Inset: Cartoon of single channel design. **B.** Top view of the image of single channel taken with fluorescent microscope. T47D cells were specifically labeled with anti-pancytokeratin antibody and Alexa 594-conjugated secondary antibody (red). HMF were specifically labeled with anti-vimentin antibody and Alexa 488-conjugated secondary antibody (green) (4x objective). **C.** Side view of the integrated Z-series image stack images taken with confocal microscope (20x objective).

Microchannel three-dimensional collagen gel co-culture was established based on a previously described conventional 3D collagen gel co-culture [13]. Microchannel culture devices (iuvo Microchannel 5250) were generously provided by BellBrook Labs (Madison, WI). T47D cells and HMF cells or primary breast fibroblasts were mixed at a ratio of 2∶1 in collagen type I (final collagen concentration of 1.3 mg/ml.) 1.5 µl of the cell suspension in collagen were loaded in each channel, resulting in approximately 800 cells per channel. All fluid changes were accomplished using surface tension effects (passive pumping; **Fig. 1A** **inset**) [29]. Limited gel retraction from the roof of the channel (height approximately 140 µm) creates a gap that allows efficient pumping from input to output port [30]. The loaded microchannel device was kept in a moisturized bioassay container and incubated at 37°C in a humidified atmosphere containing 5% CO_2_ for 3 to 5 days. For the neutralizing antibody treatments, antibody was added to both collagen gel and media at the final concentrations indicated in **Table S1**.

### Immunofluorescence Staining of Microchannel Collagen Gel Co-culture

Collagen gel co-cultures grown in microchannels were rinsed, fixed and permeablized with 0.1% Triton X-100 for 1.5 hours. After thorough PBS washes, cells were blocked and then incubated with primary antibodies at 4°C overnight. Following PBS washes, secondary antibody (1∶150) was added for an overnight incubation. After additional washes with PBS, mounting medium (90% glycerin in 100 mM TrisHCl) was added to input and output ports.

### T47D Cell and HMF Growth Assay in Microchannels

Collagen gel cultures were grown for 3 to 5 days, then fixed and stained as described above. T47D cells were labeled with anti-CK antibody. Immunofluorescence images were acquired with an NIS-Elements imaging system on an inverted microscope (Nikon Eclipse Ti). An objective with 4x magnification, which covers the total area of one microchannel, was used to acquire the images. The CK-positive area was measured using ImageJ software [31] and used as readout for T47D cell growth. Co-culture-induced T47D cell growth was calculated as: (CK-positive area of co-culture – CK-positive area of T47D monoculture) ÷ CK-positive area of T47D monoculture×100%. HMF were labeled with anti-human vimentin antibody; and nuclei were stained with Hoechst 33342 (Invitrogen). HMF were manually counted on acquired images as the number of nuclei within vimentin-positive cell bodies.

### siRNA Transfection

SiRNA oligonuleotides (ON-TARGET plus SMARTpool siRNA) were purchased from Dharmacon RNAi Technologies (Lafayette, CO). 100 nM of siRNA oligonucleotides were delivered to 3×10^4^ primary fibroblasts using Dharmacon lipid 3 (Dharmacon) transfection reagent according to the manufacturer’s protocol. 72 hours post transfection, the primary fibroblasts were lifted in trypsin (0.25% wt/vol) and co-cultured with T47D cells in collagen gels. The efficacy of siRNA oligonucleotides was validated by qRT-PCR.

### Statistical Analysis

Data are presented as mean ± SE from at least three independent experiments. In each experiment, every data point was calculated as the average of 3–6 replicates. Student’s t test was applied to analyze the differences of the treated group vs. control group. P values of ≤0.05 were considered statistically significant. To analyze the heterogeneity of T47D response to the antibody treatment in co-culture with CAF or NF, the relative treatment effect was first calculated (co-culture-induced T47D cell growth with antibody ÷ co-culture-induced T47D cell growth without antibody). Subsequently, the inter-subject variances of the relative treatment effect were calculated. F-test was performed to analyze the differences of the inter-subject variance of each antibody for the group of NF, CAF-low grade, and CAF-high grade. P values of ≤0.05 were considered statistically significant.

## Results

### Characterization of Fibroblast-carcinoma Cell 3D Co-culture in Microchannel Devices

We have previously established a 3D collagen gel co-culture system and analyzed the growth regulation of T47D breast cancer cells in the presence of human mammary fibroblasts (HMF) in conventional tissue culture plates [13], [26] and in microchannel devices [32]. Therefore, we initially used the same cell lines to characterize the microchannel co-culture platform used in this study. Collagen polymerization conditions for HMF in microchannel have recently been optimized [30]. The device used in this study consists of a polystyrene plastic plate containing an array of 192 microchannels, harboring a volume of 0.7 µL each (**Fig. 1A**). HMF and T47D cells co-cultured within the micro-channels grew normally and distributed evenly along the horizontal (**Fig.** **1B**) and vertical (**Fig. 1C**) dimension of the channel. Since our previous study had demonstrated a linear correlation between the area occupied by cytokeratin (CK)-positive T47D cell clusters and the number of cells [32], we used CK immunolabeling as readout for T47D cell growth.

**Figure 2:**
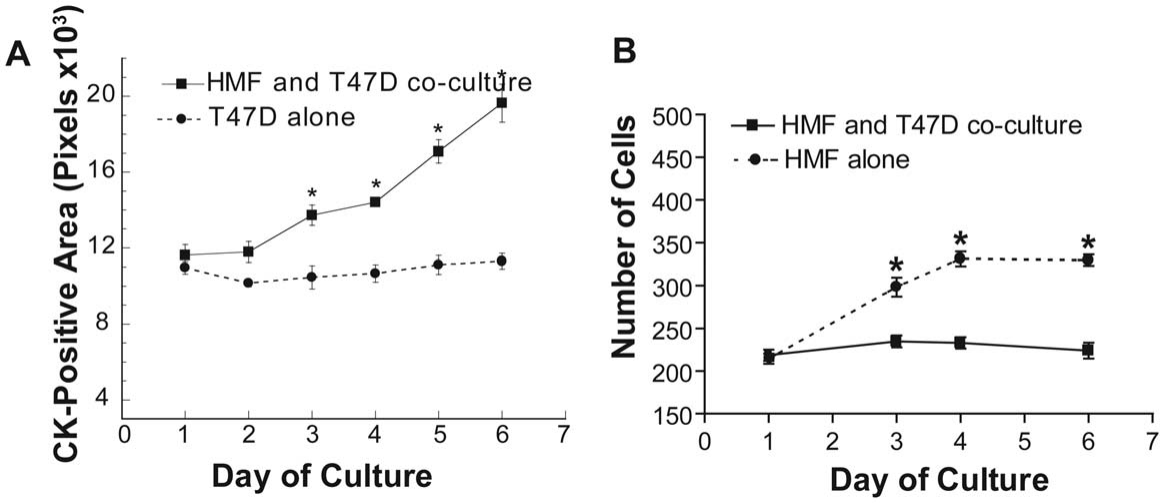
Characterization of T47D and HMF growth in micro-channel collagen gel co-culture system. A. T47D cell growth is significantly induced in co-culture with HMF compared to mono-culture. Co-culture or mono-culture gels were fixed and stained at day 1 to 6. The cytokeratin-positive area was measured with Image J. **B.** HMF proliferate at a significantly higher rate in mono-culture than in co-culture with T47D cells.

We then characterized the growth of T47D cells and HMF in the channels. Consistent with conventional co-culture, T47D cell growth was significantly induced by the presence of HMF (**Fig. 2A**) in a dose-dependent manner (**Fig. S1A, B**). The HMF-mediated growth advantage of T47D cells became significant after three days of culture. In contrast, HMF grew slower in co-culture with T47D cells than in monoculture (**Fig. 2B**). The finding of fibroblast growth inhibition by epithelial cells is consistent with observations reported by other groups [33]. HMF-dependent T47D cell growth stimulation was maintained in compartmentalized, non-contact co-culture but decreased with a widening distance between the epithelial and fibroblast compartments, strongly suggesting that diffusible, paracrine factors are responsible for promoting mitogenesis (**Fig. S1C**).

### Identification of Paracrine Signaling Factors Required for HMF-induced T47D Cell Growth in 3D Collagen Co-culture

To identify the paracrine factors responsible for T47D carcinoma cell growth stimulation we treated the co-cultures with neutralizing antibodies to eleven factors implicated in stroma-carcinoma interactions (FGF-2, HB-EGF, Heparanase-1, HGF, IGF-1, IGF-2, MT1-MMP, PDGF, SDF-1, TGF-β1, and Wnt-1). To uncover potential co-culture-independent effects, we first tested the neutralizing antibodies in T47D cell and HMF monoculture. Anti Wnt-1 antibody significantly inhibited both T47D cell and HMF growth in monoculture (**Fig. S2A & B**). Since the focus of this study was on paracrine signaling in co-culture, we excluded the anti-Wnt-1 antibody from further consideration. The other ten antibodies did not affect either T47D cell or HMF growth in monoculture.

**Figure 3:**
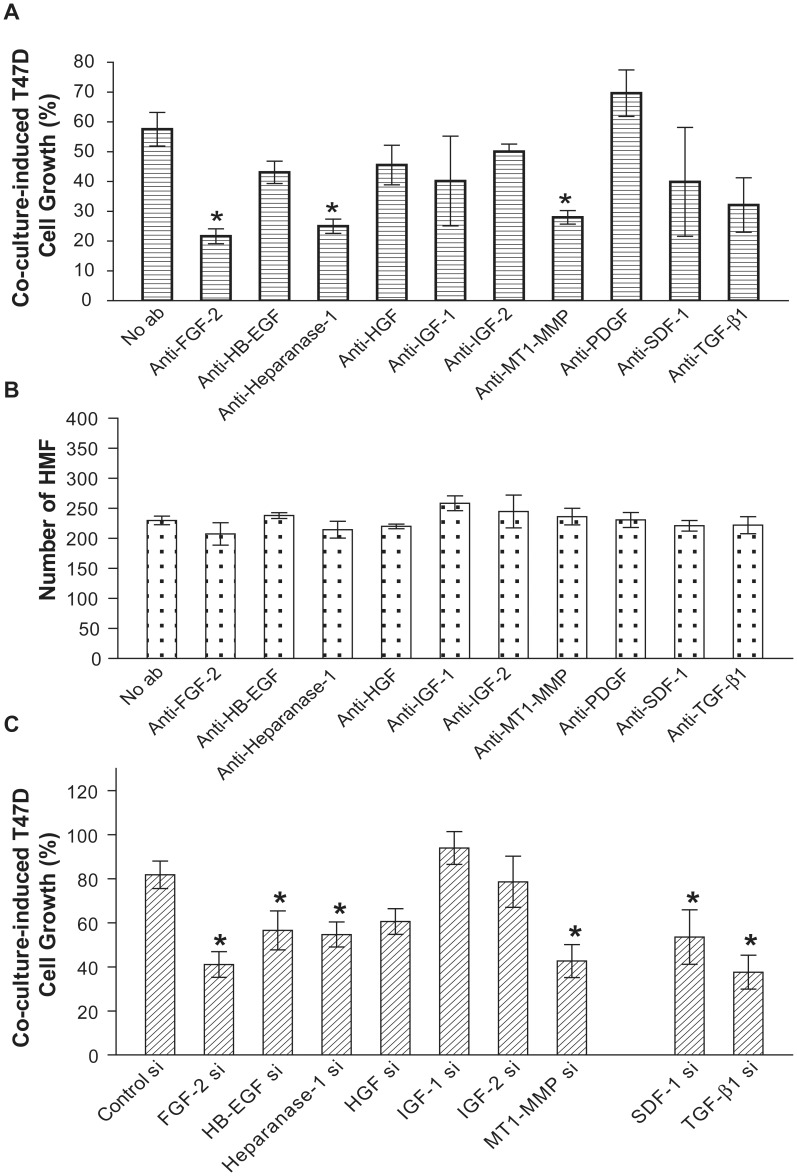
Identification of paracrine signaling factors regulating T47D cell growth in co-culture with HMF. A. In co-culture with HMF, T47D cell growth is significantly reduced by neutralizing antibodies against FGF-2, Heparanase-1, or MT1-MMP. Neutralizing antibodies (see **Table S1** for details) were added to collagen gel and culture media. Co-cultures were fixed and stained after 3–4 days of treatment. Co-culture-induced T47D cell growth was calculated as described in Materials and Methods. **B.** Neutralizing antibodies do not affect HMF growth in co-culture. **C.** T47D cell growth was significantly decreased in co-culture with HMF transfected with siRNA targeting FGF-2, HB-EGF, Heparanase-1, MT1-MMP, SDF-1, or TGF-β1. HMF were transfected with siRNA oligonuleotides (100 nM) 3 days before co-culture with T47D cells. Co-cultures were incubated for 3 days, then fixed and stained. Data shown represent the mean of 3 independent experiments. In each experiment, 3–6 micro-channels were used as technical replicates for every group. Student’s t-test was applied to compare specific treatment vs. no antibody control. The asterisk denotes P<0.05.

Neutralizing antibodies to FGF-2, Heparanase-1, and MT1-MMP significantly reduced T47D cell growth in co-culture with HMF (**Fig. 3A**). None of the neutralizing antibodies affected HMF growth in co-culture (**Fig. 3B**). Therefore, the reduction of T47D cell growth in the presence of neutralizing antibodies could not be simply attributed to lower HMF numbers. Instead, this result suggests that FGF-2, Heparanase-1 and MT1-MMP regulate T47D cell growth as participants in the paracrine milieu.

To further examine the paracrine signaling function of these mediators, and to identify the source of these factors in co-culture, we silenced their expression in HMF by siRNA treatment prior to combining the fibroblasts with T47D cells in the channels. Since PDGF exists in multiple isoforms and because siRNA oligonuleotides that effectively target all of its isoforms are not available, we were not able to include PDGF in this RNAi screen. The efficacy of the siRNA oligonuleotides was confirmed by quantitative RT-PCR (**Fig. S3**). IGF-1 and IGF-2 mRNA was undetectable in HMF. T47D cell growth was significantly suppressed in co-culture with HMF transfected with siRNA targeting FGF-2, HB-EGF, Heparanase-1, MT1-MMP, SDF-1, or TGF-β1 (**Fig. 3C**). This further supports a growth-promoting role of FGF-2, HB-EGF, Heparanase-1 and MT1-MMP and indicates that fibroblasts are the principal source. The siRNA results also suggest that in addition, fibroblast-derived SDF-1 and TGF-β1 are required for T47D cell growth stimulation in co-culture. These observations are consistent with our prior finding that FGF-2, SDF-1 and MT1-MMP are required for fibroblast-dependent T47D cell growth stimulation using a conventional co-culture system [13], [26].

### Identification of Paracrine Signaling Factors Required for Breast Carcinoma Cell Growth Stimulation by Primary Stromal Fibroblasts

**Figure 4:**
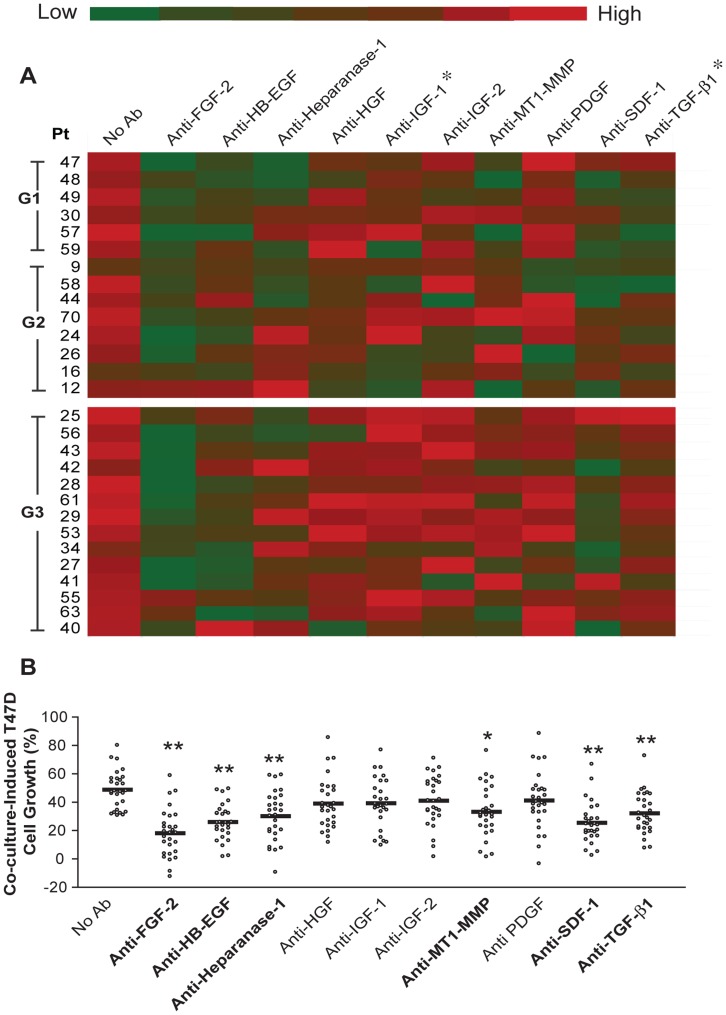
Functional screen of paracrine factors in co-culture of CAF with T47D cells. A. Red-green heat map representation of CAF co-culture-induced T47D cell growth in the presence or absence of neutralizing antibodies. Antibody treatment and calculation of Co-culture-induced T47D cell growth were performed as described in the legend of **Fig. 3**. Color changes from green to red as value increases. Each row depicts data for CAF from an individual patient (Pt number on left). The tumor grade is indicated on the left-hand side of Pt number. Cases are stratified into low grade (G1 and G2) and high grade (G3). Asterisk denotes neutralizing antibodies, where significant differences were detected between low-grade and high-grade cancer group. Each data point represents the mean of 3–6 replicates. **B.** Scatter plot representation of the data shown in panel “**A**”. Student t-test was applied to compare specific treatment vs. no antibody control. * P = 0.0006, ** P<0.0001.

We next investigated paracrine signaling interactions between breast carcinoma cells and primary fibroblasts isolated from human tissue samples. Carcinoma associated fibroblasts (CAF) were isolated from human breast carcinoma resection specimens. Normal mammary fibroblasts (NF) were obtained from adjacent normal breast tissue. All tissue samples were examined microscopically to confirm the presence or absence of carcinoma and to determine tumor subtype and pathologic grade of the carcinomas. The distribution of subtypes, grade, hormone receptor and Erb-B2 overexpression status were representative for breast carcinomas in the general population (**Table S2**). CAF and NF were then grown in 3D co-culture with T47D cells and paracrine interactions were screened with neutralizing antibodies as described for HMFs. When evaluating all cases collectively, blocking antibodies to FGF-2, HB-EGF, heparanase-1, MT1-MMP, SDF-1 and TGF-β1 significantly diminished carcinoma cell growth stimulation (**Fig. 4A, B**) and this effect was antibody dose-dependent (**Fig. S4A–G)**. However, the CAF from individual tumors showed a considerable degree of heterogeneity and some did not fit into this general pattern (**Fig. 4A**). For example, in contrast to the majority of cases, T47D co-cultures with CAF from Patient 41 did not respond to neutralization of SDF-1 or MT1-MMP activity, but to blocking of PDGF and IGF-2.

The paradigm that transformed tumor cells and host stromal cells co-evolve during tumor progression is increasingly gaining recognition. Pathologic grade is a generally accepted measure of the differentiation or progression state of the carcinoma cell component of the tumor. We postulated, that the degree of aggressiveness, assessed by grade, would be reflected in the CAF, and result in different paracrine fibroblast-carcinoma cell interactions. Indeed, we found that 50% and 64% of co-cultures with CAF from low-grade (grade 1 or 2) carcinomas responded to antibodies blocking IGF-1 and TGF-β1, respectively, whereas all co-cultures with CAF from high-grade (grade 3) carcinomas failed to respond to these inhibitors (**Fig. 4A** **, Table S3**). Immunohistochemical labeling of the carcinoma tissue samples in TMA demonstrated IGF-1 and TGF-β1 expression by both carcinoma cells and stromal fibroblasts in many of the tumors (**Fig. S5**) but failed to detect a correlation between response to inhibitors in co-culture and growth factor labeling in the stromal fibroblasts. This observation indicates that response to the inhibitors is not merely related to stromal growth factor levels but instead suggests that CAF from high-grade carcinomas have become resistant to these inhibitors and utilize alternative paracrine pathways to stimulate carcinoma growth. One of the defining characteristics of breast cancer is the presence or absence of steroid hormone receptors in the carcinoma cells. Therefore, we examined whether hormone receptor status of the carcinoma cells impacts on the paracrine signaling network of the fibroblasts isolated from these tumor. Inhibition of HB-EGF significantly diminished carcinoma cell growth in co-cultures with CAF from all eight hormone receptor negative (ER negative or PR negative) carcinomas but only in 44% of co-cultures with CAF from hormone receptor positive (ER and PR positive) tumors (*P* = 0.0095; **Table S3**). None of the other paracrine mediators showed any association with the hormone receptor status of the primary carcinomas.

**Figure 5:**
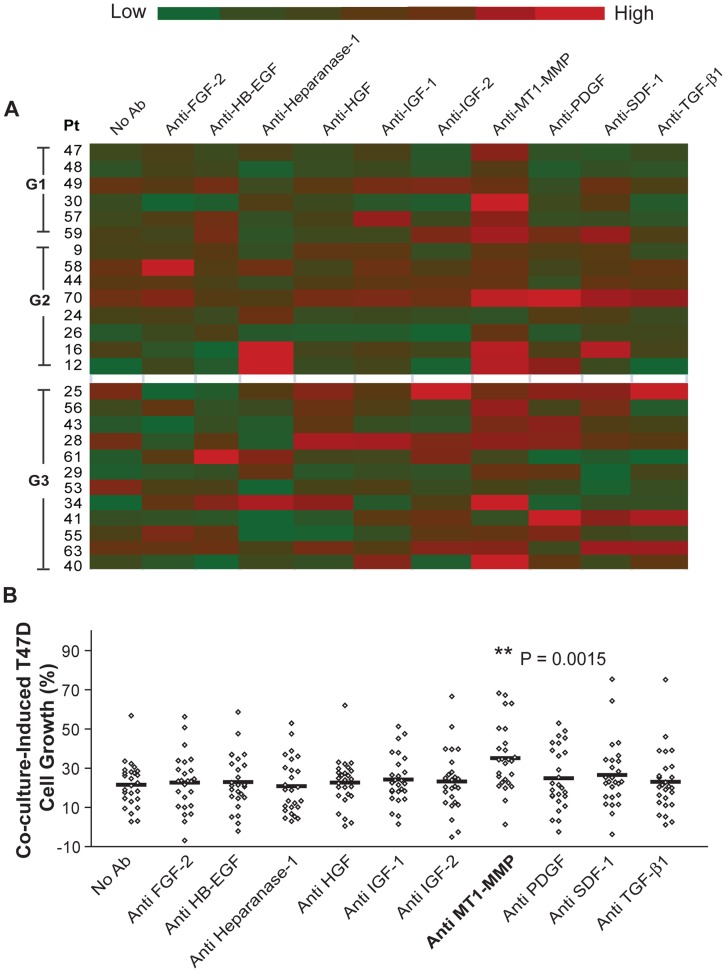
Functional screen of paracrine factors in co-culture of NF and T47D cells. A. Red-green heat map representation of NF co-culture-induced T47D cell growth in the presence or absence of neutralizing antibodies. Antibody treatment and calculation of Co-culture-induced T47D cell growth were performed as described in the legend of **Fig. 3 and 4**. Color changes from green to red as value increases. Each row depicts data for NF from an individual patient (Pt number on left). The tumor grade is indicated on the left-hand side of Pt number. Cases are stratified into low grade (G1 and G2) and high grade (G3). **B.** Scatter plot representation of the data shown in panel “**A**”. Student t-test was applied to compare specific treatment vs. no antibody control. Each data point represents data of one NF sample.

NF stimulate T47D cell growth in co-culture as well, albeit to a significantly lesser degree than CAF (**Fig. S6**). This observation is consistent with our previous study using conventional 3D co-culture [26]. Collectively, none of the neutralizing antibodies reduced T47D cell growth in co-culture (**Fig. 5A, B**). Surprisingly, a function-blocking antibody to MT1-MMP significantly stimulated T47D cell growth in co-culture (**Fig. 5B**). This growth stimulation was dose-dependent at lower concentrations and reached a plateau or showed a biphasic pattern at higher antibody concentrations (**Fig. S4H**). The opposing functions of MT1-MMP in CAF vs. NF support the concept that paracrine interactions are highly context-dependent and that growth inhibitory signals in normal tissues may become mitogenic during malignant transformation.

**Figure 6:**
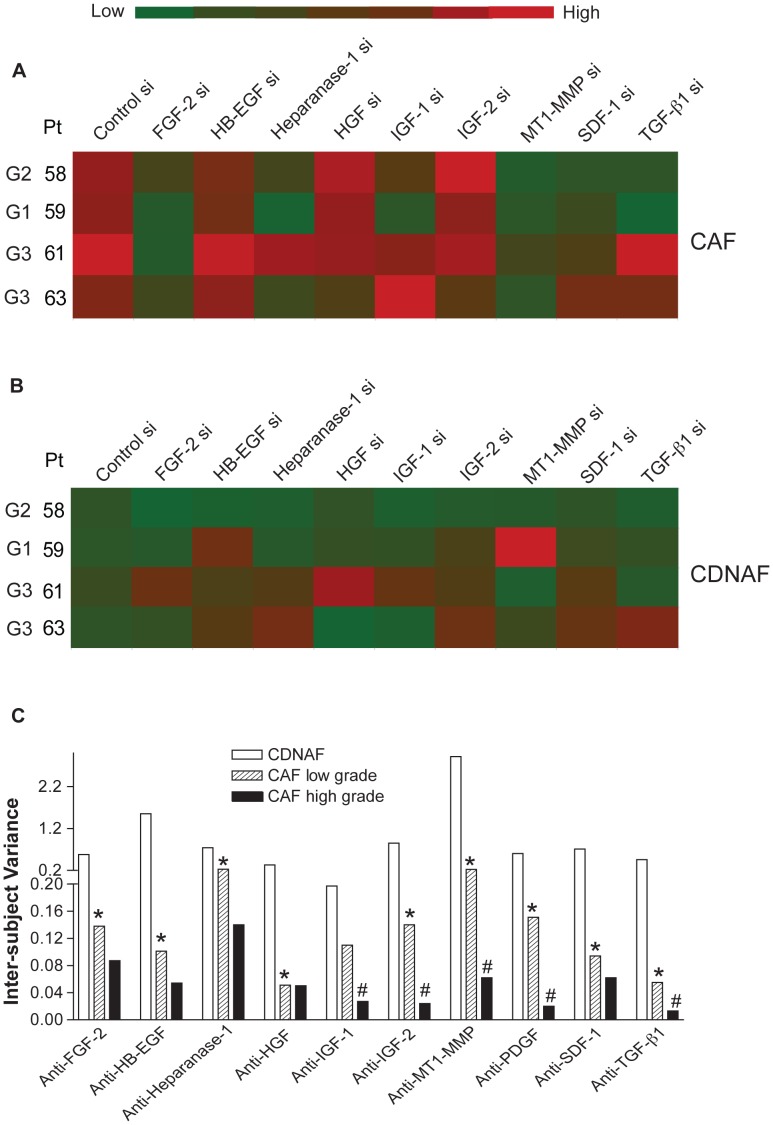
siRNA screen of T47D cell growth in co-culture with CAF or NF and inter-subject heterogeneity of paracrine interactions. A & B . Red-green heat map representation of T47D cell growth stimulation in co-culture with CAF (**A**) or NF (**B**). SiRNA oligonuleotide transfection was performed as described in Materials and Methods to knock down expression of specific mediators. Co-culture-induced T47D cell growth was calculated as described for Fig. 3. Each data point represents the mean of 3–6 replicates. **C**. Inter-subject heterogeneity of T47D cell growth response to neutralizing antibody is highest in co-culture with NF and lowest in co-culture with CAF from high-grade tumors. Co-culture-induced T47D cell growth in the presence of antibody was normalized to the no-treatment control for each patient. The sample variance for the group of NF, CAF low-grade, or CAF high-grade were then calculated. F test was applied to compare variances between the groups. * P<0.05, CAF of low grade tumor vs. NF, # P<0.05, CAF of high grade tumor vs. CAF of low grade tumor.

The addition of a blocking antibody effectively neutralizes the activity of the targeted paracrine factor in the microenvironment. To gain additional insight into the cell source of the targeted factors and to validate “hits” with an alternative approach, we used RNAi technology to knock down expression of the paracrine factors in primary fibroblasts prior to co-culture. We selected four pairs (CAF and NF) of primary cultured fibroblasts to encompass all tumor grades. The efficacy of the siRNA oligonuleotides was validated by quantitative RT-PCR, demonstrating 80–99% reduction of the mRNA targets in CAF and NF (**Fig. S7A & B**). In contrast to HMF, both CAF and NF expressed IGF-1. In the majority of CAF samples, T47D cell growth stimulation was attenuated by siRNA targeting FGF-2, Heparanase-1, MT1-MMP, and SDF-1 (**Fig. 6A**). Consistent with the antibody screen, knock-down of IGF-1 or TGF-β1 expression suppressed T47D cell growth only in co-cultures with CAF from low-grade tumors. siRNA knock-down of MT1-MMP in NF of patient 59 significantly stimulated T47D cell growth in the co-culture, confirming the neutralization experiment and identifying the fibroblasts as the production site of this enzyme (**Fig. 6B**). Overall, the pattern of T47D growth inhibition was similar for individual fibroblast samples used in the co-culture - regardless of the method applied to suppress the activity of the paracrine factor target (**Fig. 4** **,** **5** **, & 6**). This finding indicates that many of the factors examined here are produced by the fibroblasts and further supports the concept that carcinoma aggressiveness is mirrored by functional characteristics of the stromal fibroblasts.

We then analyzed by F-test the inter-subject variance of the T47D cell response in co-culture with NF or CAF. We found that the T47D cell response in co-culture with NF had a significantly greater variance than in co-culture with CAF (**Fig. 6C**), suggesting that carcinoma cell growth stimulation by CAF is more uniform than by NF. Interestingly, in co-cultures with CAF from high-grade tumors, the inter-subject variance was significantly smaller than in co-culture with CAF from low-grade tumors (**Fig. 6C**). These findings suggest that mammary fibroblasts gradually lose the inter-subject heterogeneity and become functionally more homogeneous as tumors develop and progress to a more aggressive phenotype.

## Discussion

Using loss-of-function screens in 3D co-cultures of primary fibroblasts from 28 breast cancer patients with a breast carcinoma cell line, we have identified paracrine, secreted factors that regulate carcinoma cell mitogenesis. This screen, which was made feasible by a recently developed microfluidic culture platform [30], [32], [34], uncovered a functional patient-to-patient heterogeneity of paracrine mediators. Despite this heterogeneity, FGF-2, HB-EGF, Heparanase-1 and MT1-MMP, emerged as paracrine growth stimulators active in co-cultures with most CAF samples. IGF-1 and TGF-β1 were required for growth stimulation by CAF from low-grade but not high-grade carcinomas, indicating a grade-dependency of paracrine signaling pathways.

To limit the number of variables and focus on the patient-to-patient variability of the fibroblasts, we used one cell line as indicator to represent the carcinoma component in the co-cultures. T47D cells are considered relatively well differentiated and fall into the common category of luminal type, ER, PR positive and Her-2 negative [35]. Naturally, the paracrine factors identified by the functional screen depend on the expression of growth factor receptors by T47D cells and the functionality of signaling pathways downstream of these receptors. For example, T47D cells express only low levels of the receptor tyrosine kinase Met [36], which explains why the neutralizing antibody targeting its cognate ligand HGF had little effect in co-culture with CAF. We plan to expand the co-culture screens to include other breast carcinoma subtypes and primary, stroma-matched patient-derived carcinoma cells.

In agreement with our previous work on immortalized mammary fibroblasts, FGF-2 was identified as important paracrine growth promoting factor in CAF from all but three carcinomas. siRNA expression silencing confirmed that FGF-2 originates from the fibroblasts but it is unclear whether the growth factor acts on the carcinoma cells or modulates the fibroblasts in an autocrine manner. A direct stimulatory effect of FGF-2 on T47D cells has been reported by us [13] and others [37], however, it is likely that an indirect modulation of CAF behavior by FGF-2 also plays a role. Approximately 10% of all and 16–23% of luminal type B breast carcinomas show FGFR1 gene amplification; one of the most common focal amplifications observed in breast cancer [38], [39]. Amplification is tightly linked to FGFR1 overexpression in patient samples and forced overexpression of FGFR1 drives resistance to hormonal therapy in vitro [39].

SDF-1 is well established as an important fibroblast-derived paracrine factor that promotes breast carcinoma cell growth in vitro and in vivo by direct paracrine stimulation of carcinoma cells and by stimulating angiogenesis [9]. Heparanase-1, an endoglucoronidase, cleaves heparan sulfate glycosaminoglycans in the extracellular matrix and at the cell surface into smaller fragments [40]. In human breast carcinoma, heparanase-1 expression is associated with larger tumor size and lymph node metastasis [41]. Several modes of action have been proposed: Heparanase-1 releases growth factors from their heparan sulfate storage sites in the ECM and generates smaller, bioactive heparan sulfate fragments, which may enhance growth factor – receptor interactions at the cell surface. Heparanase-1 also enhances proteolytic shedding of the proteoglycan syndecan-1 from the cell surface (see below) [13], [26]. Our experiments also reveal TGF-β1 as carcinoma growth promoter in co-cultures of CAF from low-grade carcinomas, which reflect the molecule’s complex and context-dependent function in cancer [42].

Hotary et al. showed that the enzyme MT1-MMP stimulates the growth of carcinoma cells embedded in 3D collagen but not in 2D monolayer culture - an activity that requires collagen degradation [43]. In human breast carcinomas, MT1-MMP is mostly stroma-derived [25]. We have recently shown that MT1-MMP cleaves syndecan-1, a proteoglycan induced in stromal fibroblasts by neighboring carcinoma cells in vivo and in vitro [26]. Sdc1 ectodomain, thus released from the fibroblast cell surface, can act as paracrine growth stimulator in concert with FGF-2 and SDF-1 [13]. Paradoxically, in co-culture with NF, MT1-MMP appears to act as a growth inhibitor of carcinoma cells. A similar tumor suppressor activity has been proposed for MMP 3, 8, 9 and 12 [44]. The simplest explanation for the opposite effects seen with CAF and NF would be that the fibroblast types produce different substrates. This hypothesis is plausible considering that a differential proteomics screen has described a large and diverse group of potential MT1-MMP substrates – many of which are candidate paracrine signaling molecules [45]. These findings indicate that similar to TGF-β1, MT1-MMP possesses dual functions as growth suppressor and stimulator and that these activities are critically regulated by the micro-environmental context. The paradoxical activities of MT1-MMP also offer an attractive explanation for the failure of MMP inhibitors in clinical trials. Two clinical trials had to be aborted because the MMP inhibitor led to accelerated tumor progression [46], [47].

One surprising finding of our study is the fact that loss of function of any single one of these factors (FGF-2, HB-EGF, heparanase-1, SDF-1 or TGF-β1) reverses the growth advantage imparted by CAF. This apparent simultaneous dependency on multiple paracrine factors suggests a complex network of interactions between stromal fibroblasts and carcinoma cells. This apparent addiction to stromal signals creates cautious optimism that a therapeutic disruption of these pathways might retard breast carcinoma growth. A microfluidic-based high-throughput co-culture assay platform as described here, could be used to identify the critical factors in individual patients and customize stroma-targeted therapy.

The stromal tumor compartment in breast carcinomas is characterized by a remarkable inter-individual heterogeneity of gene expression [6]. The stromal expression signatures can be used to cluster individual tumors into distinct subclasses, which stratify patients into prognostic groups [12]. Our study demonstrates that this heterogeneity extends to functional activities of stromal fibroblasts. The fibroblast diversity may be the result of differences in stromal cell composition or due to differences in paracrine induction (e.g. trans-differentiation) by adjacent carcinoma cells [48]. Differences in stromal fibroblast composition may be caused by variations in the recruitment of fibroblast precursors (e.g. local recruitment of resident fibroblasts vs. marrow-derived mesenchymal stem cells) or by selection of fibroblast subtypes in the tumor microenvironment. Unexpectedly, we observed a trend towards a loss of functional heterogeneity of mammary fibroblast as tumors develop and then progress into a more malignant type. This observation is consistent with our previous finding that global gene expression is more variable in NF than in CAF [6].

In summary, functional screens of paracrine fibroblast-carcinoma signaling networks may provide us with the understanding necessary to design rational, stroma-targeted therapies which disrupt these signaling pathways. The present study represents a step in this direction.

## Supporting Information

Figure S1 **Co-culture-induced T47D cell growth is HMF dose and distance-dependent. A.** The number of T47D cells in co-culture was kept constant at 600 cells per channel. The number of HMF in co-culture was increased from 300 to 3500. Co-culture-induced T47D cell growth was calculated as described in Materials and Methods. HMF-mediated T47D cell growth stimulation increases with rising HMF numbers and then gradually reaches saturation. **B.** The HMF dose effect is maintained when total cell number per channel is kept constant, indicating that increased T47D cell growth stimulation is not caused by elevated total cell numbers. The total number of cells in co-culture was maintained at 1500 cells/µl. The ratio of T47D cell and HMF in co-culture was set to 4∶1, 2∶1, 1∶1, 1∶2, and 1∶4. The number of HMF was calculated based on these ratios. Co-culture-induced T47D cell growth was calculated as described in Materials and Methods. Each data point represents the mean of 3 independent experiments. In each experiment, 6–10 micro-channels were used as technical replicates for every data point. Co-culture and mono-culture were compared using Student’s t-test. The asterisk denotes P<0.05. **C.** T47D cells and HMF were grown in compartmentalized non-contact co-culture and the distance was controlled by inserting a cell-free gel of varying thickness (“gap distance”).
(TIF)

Figure S2 **Effect of neutralizing antibodies on T47D cell and HMF growth in monoculture. A.** T47D cell monoculture was treated with neutralizing antibodies for 3–4 days, then fixed and stained. T47D cells were stained with anti-Pan-cytokeratin antibody and labeled area was quantified. **B.** HMF monoculture was treated with neutralizing antibodies for 3–4 days, then fixed and labeled with anti-vimetin antibody and Hoechst 33342 dye as nuclear counterstain. The number of HMF was determined as the number of nuclei within vimentin-positive cells. Data represent the mean of at least 3 independent experiments. In each experiment, 3–6 micro-channels were used as replicates for each treatment. Student’s t-test was applied to compare antibody treatment with no-treatment control. Asterisk indicates P<0.05.
(TIF)

Figure S3 **RNA expression knock-down by siRNA oligonucleotide treatment.** HMF were transfected with 100 nM siRNA oligonucleotides. Total RNA was extracted 4 days after transfection and qRT-PCR was performed using GAPDH as reference. Relative expression in siRNA treated cells vs. control siRNA treated cells was calculated as: 2^(CT(Control si - GAPDH) - CT(Target si - GAPDH))^×100%.
(TIF)

Figure S4 **Dose effect of neutralizing antibodies on T47D cell growth in co-culture with CAF or CDNAF. A–G.** 3–5 CAF samples were randomly selected from CAF that displayed significant inhibition by the respective neutralizing antibody. Co-cultures of CAF with T47D cells were treated with neutralizing antibody for 3 days, then fixed and labeled with anti-Pan-keratin antibody. **H.** CDNAFs from 5 different patients were selected from CDNAF samples that displayed significant T47D cell growth induction by MT1-MMP inhibition. For each CDNAF sample, co-cultures were treated with anti-MT1-MMP antibody for 3 days. Co-culture-induced T47D cell growth in the presence of antibody was normalized to the no-antibody control. Co-culture-induced T47D cell growth was calculated as (area of co-culture - area of monoculture)/area of monoculture×100% and normalized using the no-antibody control as reference. Each data point represents the mean of 3–6 replicates.
(TIF)

Figure S5 **Immunohistochemical detection of TGF-β1 and IGF-1.** Slides were prepared from a tissue microarray that contained duplicate tissue cores representing the specimens from which CAF and CDNAF had been isolated. Immunolabeling for TGF-β1 and IGF-1 was performed using polyclonal rabbit antibodies. **A.** Breast carcinoma labeled with antibody to TGF-β1. **B.** Breast carcinoma labeled with antibody to IGF-1. **C and D.** Same samples as in “A” and “B”, respectively, but the primary antibody was omitted. Abbreviations: Ca: carcinoma; F: stromal fibroblast; Original magnification: 400x.
(TIF)

Figure S6 **CAF stimulate T47D cell growth to a significantly greater degree than CDNAF.** 3D collagen co-cultures of T47D cells with CAF or CDNAF were grown for 3 or 4 days, then fixed and stained as described. T47D cells were specifically labeled with anti-human pan-keratin antibody. Student’s t-test was applied on CAF vs. CDNAF. Each data point represents one tissue sample, and was calculated as the mean of 3–6 replicates (**P<0.0001). CAF and CDNAF originating from the same patient are connected by a line.
(TIF)

Figure S7 **Knock down of target gene expression by siRNA oligonucleotide treatment.** CAF (**A**) or CDNAF (**B**) of Pt 58 were transfected with 100 nM siRNA oligonucleotides. Total RNA was extracted 4 days after transfection and qRT-PCR was performed using GAPDH as reference. The percentage of mRNA levels in siRNA treated samples vs. control siRNA treated samples was calculated as: 2 ^(CT(Control si - GAPDH) - CT(Target si - GAPDH))^×100%.
(TIF)

Figure S8 **Scatter plot representation of the data shown in** **Figure 4A** **.**
(TIF)

Figure S9 **Scatter plot representation of the data shown in** **Figure 5A** **.**
(TIF)

Table S1 **Panel of neutralizing antibodies used in the co-culture screen.**
(DOC)

Table S2 **Characteristics of carcinomas as source of CAF and NF.**
(DOC)

Table S3 **Relationship between pathologic grade or steroid hormone receptor status and T47D cell growth response to inhibiting IGF-1, TGF-â1 or HB-EGF.**
(DOC)
