# Correction: Functional Screen of Paracrine Signals in Breast Carcinoma Fibroblasts

**DOI:** 10.1371/annotation/c5015b96-51d1-4421-8b48-d2478b947959

**Published:** 2013-04-18

**Authors:** Gui Su, Kyung E. Sung, David J. Beebe, Andreas Friedl

David J. Beebe has an ownership interest in Bellbrook Labs LLC, which has licensed technology reported in this publication. This does not alter the authors' adherence to all the PLOS ONE policies on sharing data and materials. 

