# Supplementary figures and images for: Functional Screen of Paracrine Signals in Breast Carcinoma Fibroblasts
Source: PLoS One. 2012 Oct 8;7(10):e46685. doi: 10.1371/journal.pone.0046685 (PMC3466317; doi:10.1371/journal.pone.0046685)

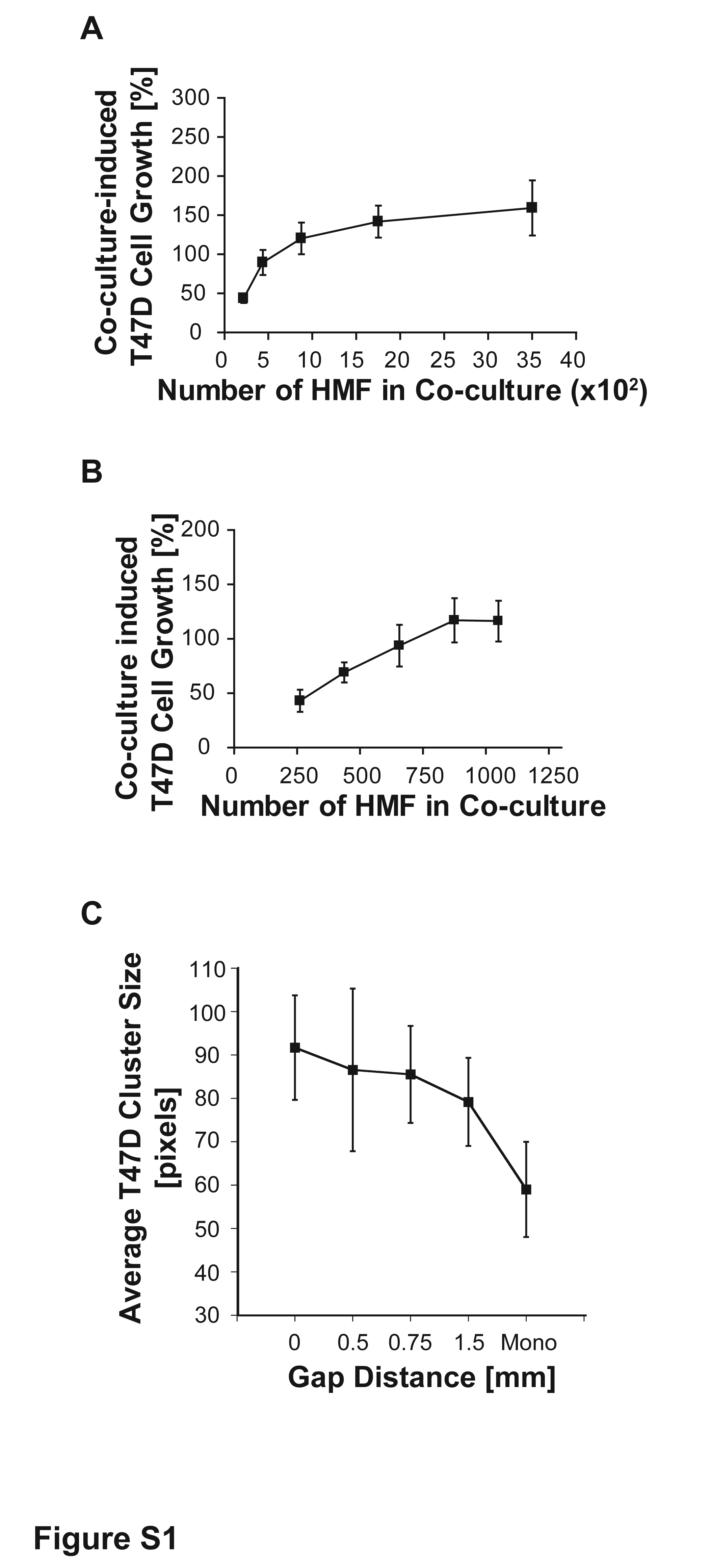

Supplement: Figure S1 — Co-culture-induced T47D cell growth is HMF dose and distance-dependent. A. The number of T47D cells in co-culture was kept constant at 600 cells per channel. The number of HMF in co-culture was increased from 300 to 3500. Co-culture-induced T47D cell growth was calculated as described in Materials and Methods. HMF-mediated T47D cell growth stimulation increases with rising HMF numbers and then gradually reaches saturation. B. The HMF dose effect is maintained when total cell number per channel is kept constant, indicating that increased T47D cell growth stimulation is not caused by elevated total cell numbers. The total number of cells in co-culture was maintained at 1500 cells/µl. The ratio of T47D cell and HMF in co-culture was set to 4∶1, 2∶1, 1∶1, 1∶2, and 1∶4. The number of HMF was calculated based on these ratios. Co-culture-induced T47D cell growth was calculated as described in Materials and Methods. Each data point represents the mean of 3 independent experiments. In each experiment, 6–10 micro-channels were used as technical replicates for every data point. Co-culture and mono-culture were compared using Student’s t-test. The asterisk denotes P<0.05. C. T47D cells and HMF were grown in compartmentalized non-contact co-culture and the distance was controlled by inserting a cell-free gel of varying thickness (“gap distance”). (TIF) [file pone.0046685.s001.tif]

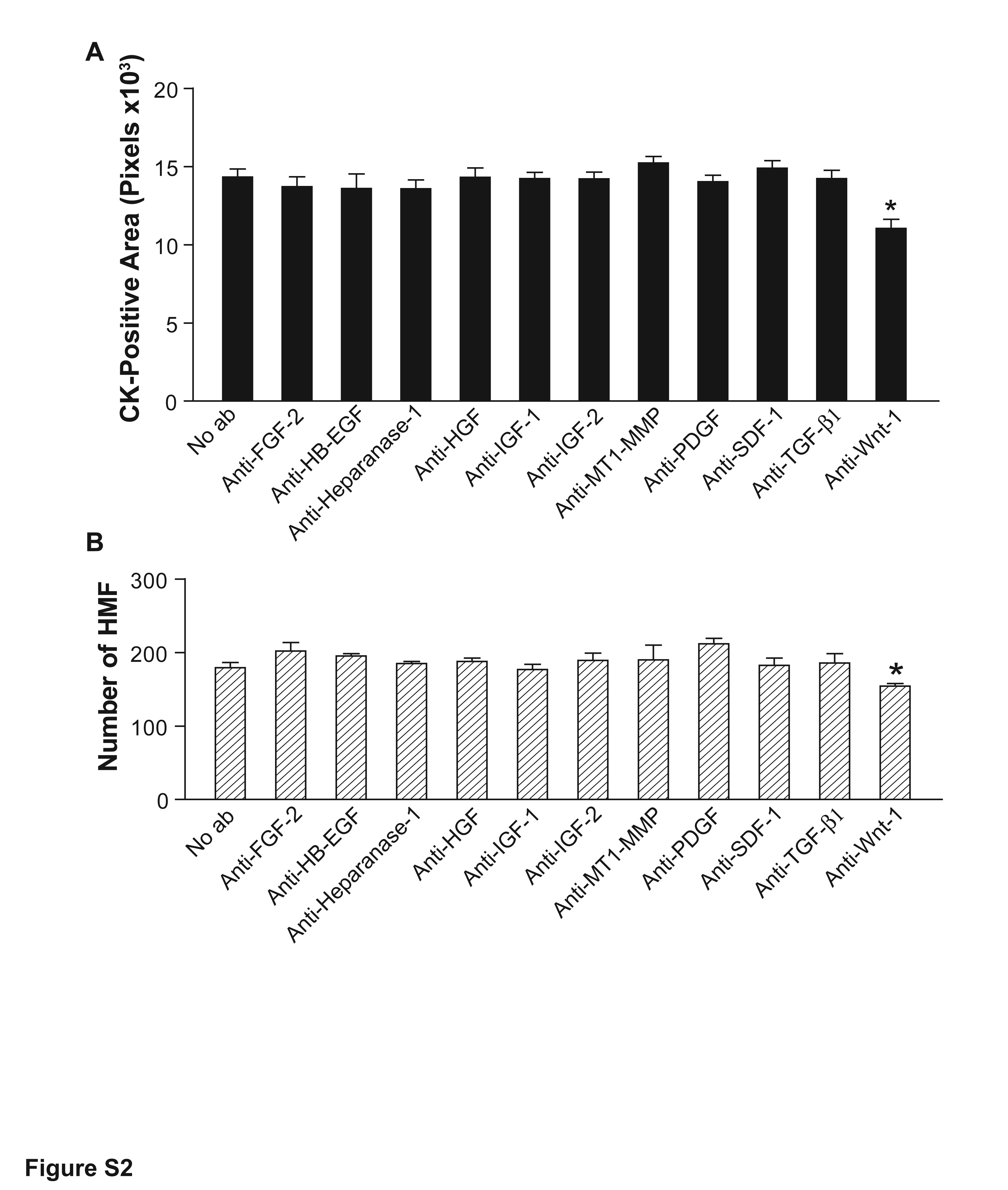

Supplement: Figure S2 — Effect of neutralizing antibodies on T47D cell and HMF growth in monoculture. A. T47D cell monoculture was treated with neutralizing antibodies for 3–4 days, then fixed and stained. T47D cells were stained with anti-Pan-cytokeratin antibody and labeled area was quantified. B. HMF monoculture was treated with neutralizing antibodies for 3–4 days, then fixed and labeled with anti-vimetin antibody and Hoechst 33342 dye as nuclear counterstain. The number of HMF was determined as the number of nuclei within vimentin-positive cells. Data represent the mean of at least 3 independent experiments. In each experiment, 3–6 micro-channels were used as replicates for each treatment. Student’s t-test was applied to compare antibody treatment with no-treatment control. Asterisk indicates P<0.05. (TIF) [file pone.0046685.s002.tif]

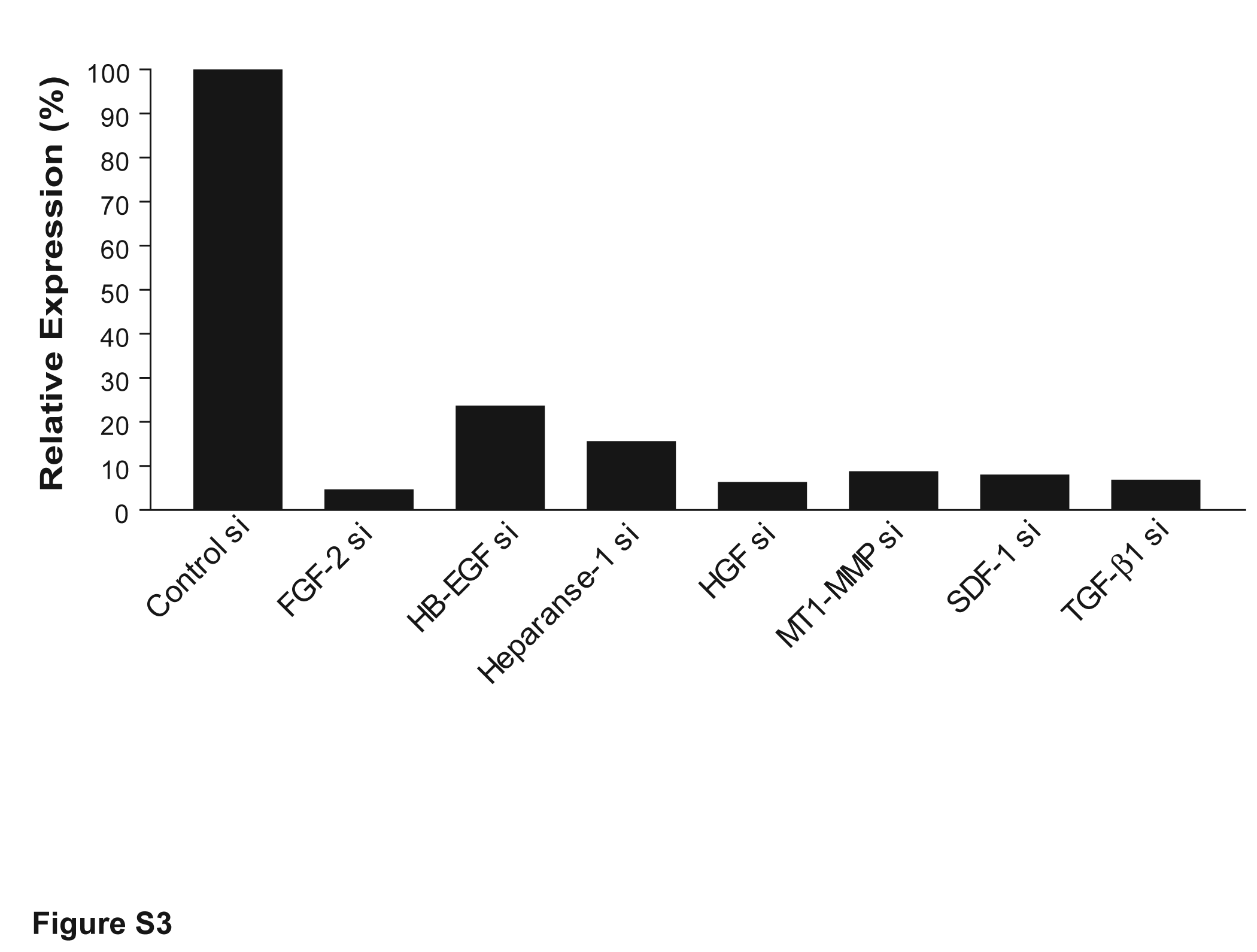

Supplement: Figure S3 — RNA expression knock-down by siRNA oligonucleotide treatment. HMF were transfected with 100 nM siRNA oligonucleotides. Total RNA was extracted 4 days after transfection and qRT-PCR was performed using GAPDH as reference. Relative expression in siRNA treated cells vs. control siRNA treated cells was calculated as: 2(CT(Control si - GAPDH) - CT(Target si - GAPDH))×100%. (TIF) [file pone.0046685.s003.tif]

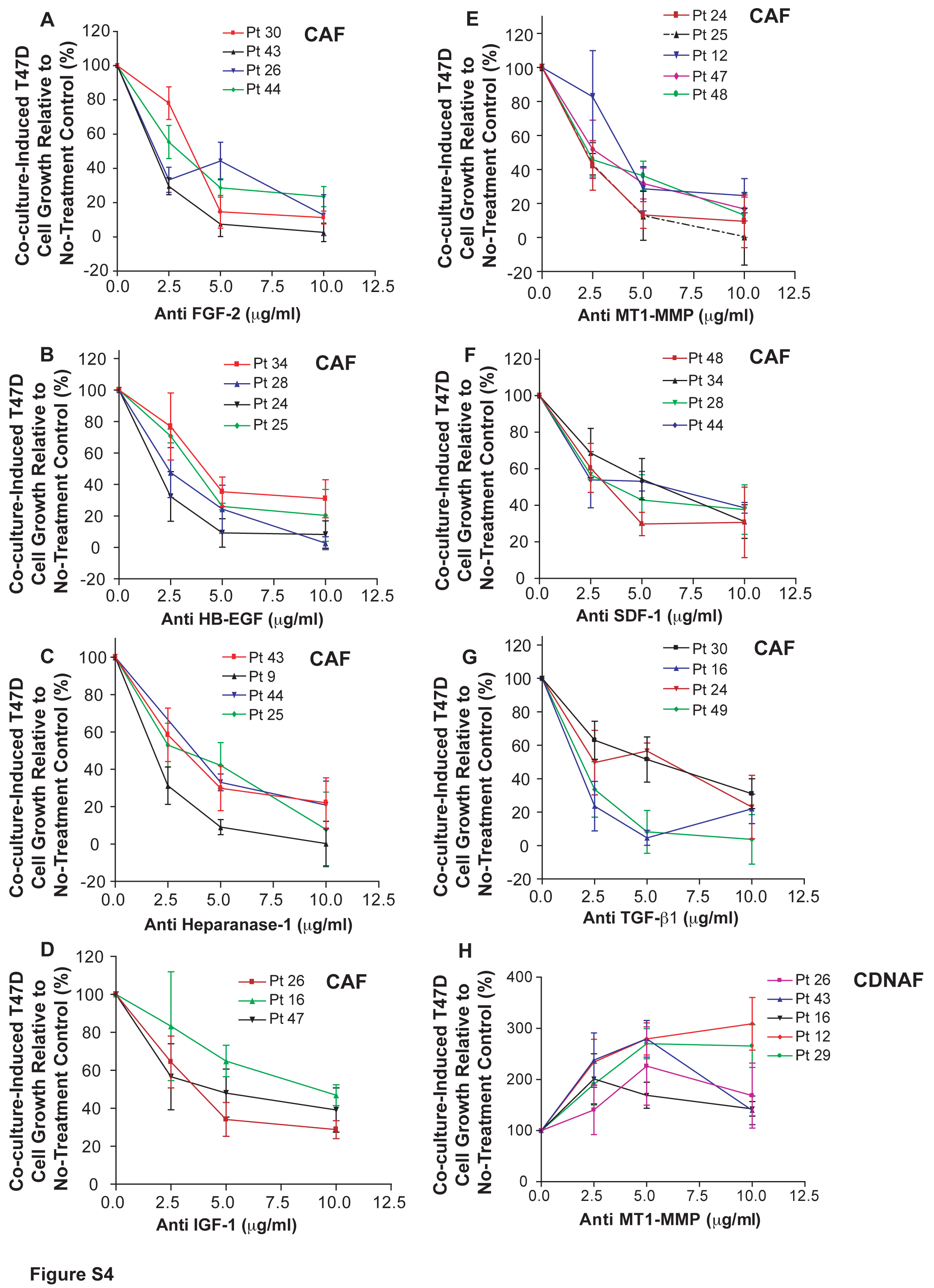

Supplement: Figure S4 — Dose effect of neutralizing antibodies on T47D cell growth in co-culture with CAF or CDNAF. A–G. 3–5 CAF samples were randomly selected from CAF that displayed significant inhibition by the respective neutralizing antibody. Co-cultures of CAF with T47D cells were treated with neutralizing antibody for 3 days, then fixed and labeled with anti-Pan-keratin antibody. H. CDNAFs from 5 different patients were selected from CDNAF samples that displayed significant T47D cell growth induction by MT1-MMP inhibition. For each CDNAF sample, co-cultures were treated with anti-MT1-MMP antibody for 3 days. Co-culture-induced T47D cell growth in the presence of antibody was normalized to the no-antibody control. Co-culture-induced T47D cell growth was calculated as (area of co-culture - area of monoculture)/area of monoculture×100% and normalized using the no-antibody control as reference. Each data point represents the mean of 3–6 replicates. (TIF) [file pone.0046685.s004.tif]

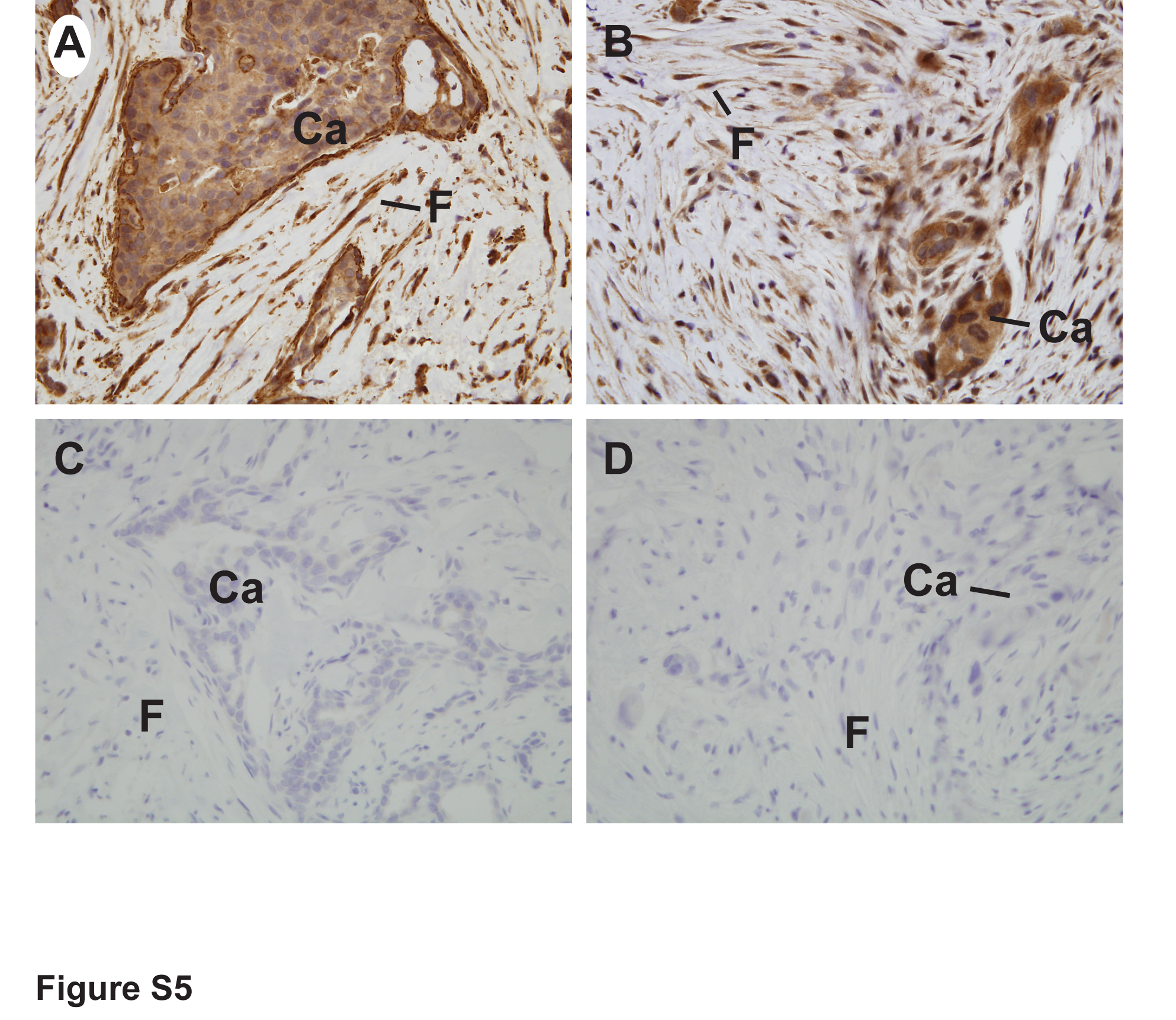

Supplement: Figure S5 — Immunohistochemical detection of TGF-β1 and IGF-1. Slides were prepared from a tissue microarray that contained duplicate tissue cores representing the specimens from which CAF and CDNAF had been isolated. Immunolabeling for TGF-β1 and IGF-1 was performed using polyclonal rabbit antibodies. A. Breast carcinoma labeled with antibody to TGF-β1. B. Breast carcinoma labeled with antibody to IGF-1. C and D. Same samples as in “A” and “B”, respectively, but the primary antibody was omitted. Abbreviations: Ca: carcinoma; F: stromal fibroblast; Original magnification: 400x. (TIF) [file pone.0046685.s005.tif]

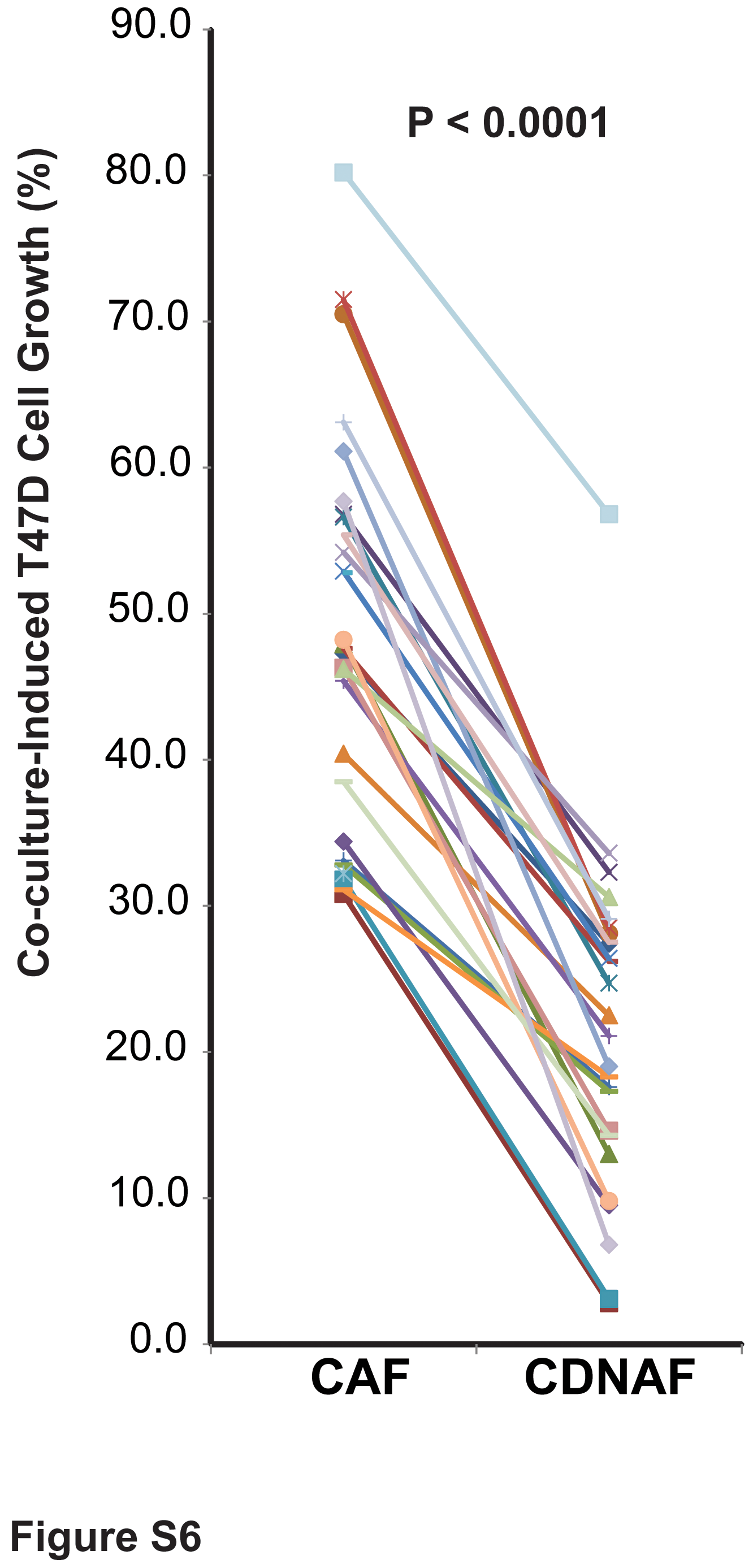

Supplement: Figure S6 — CAF stimulate T47D cell growth to a significantly greater degree than CDNAF. 3D collagen co-cultures of T47D cells with CAF or CDNAF were grown for 3 or 4 days, then fixed and stained as described. T47D cells were specifically labeled with anti-human pan-keratin antibody. Student’s t-test was applied on CAF vs. CDNAF. Each data point represents one tissue sample, and was calculated as the mean of 3–6 replicates (**P<0.0001). CAF and CDNAF originating from the same patient are connected by a line. (TIF) [file pone.0046685.s006.tif]

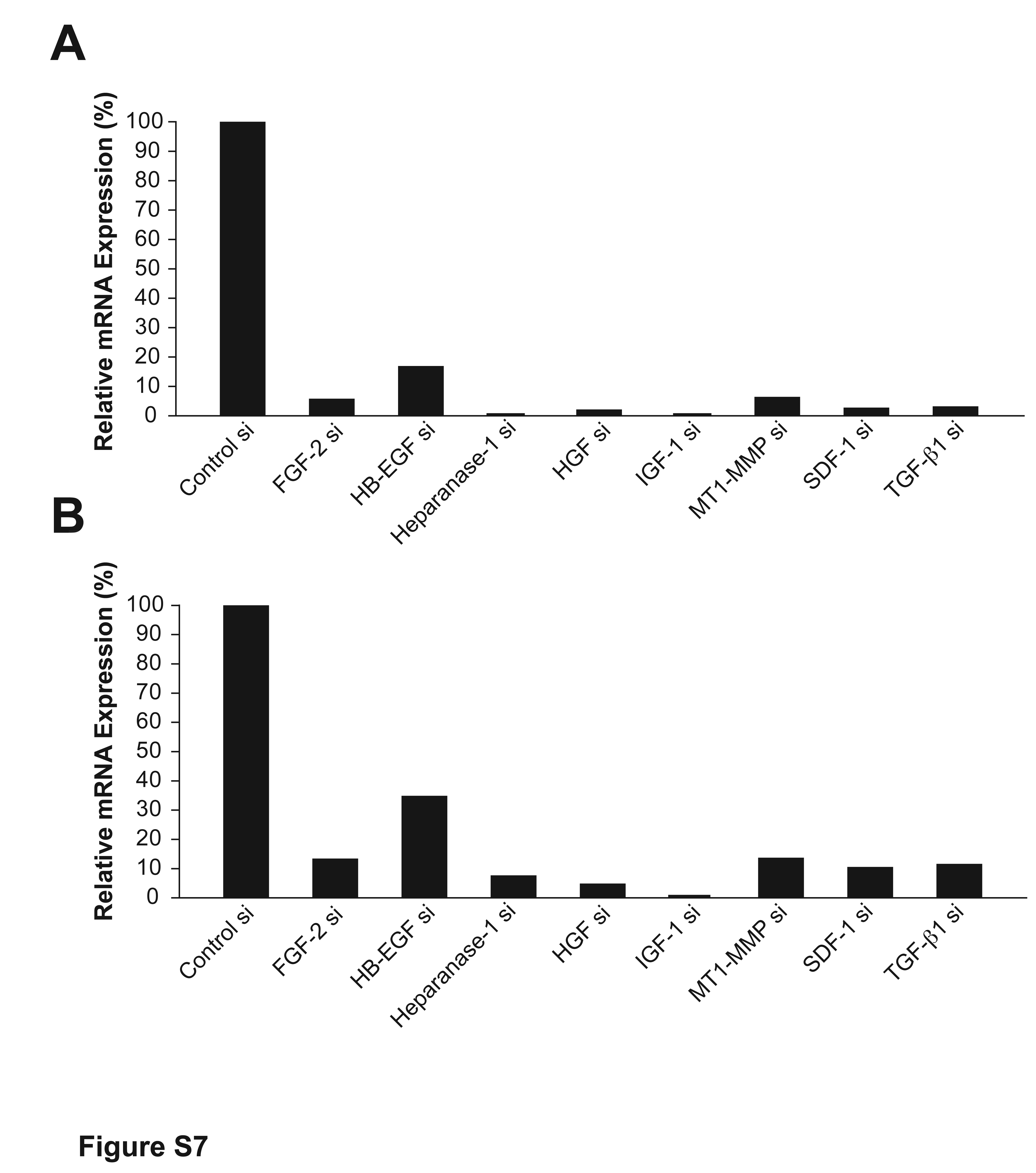

Supplement: Figure S7 — Knock down of target gene expression by siRNA oligonucleotide treatment. CAF (A) or CDNAF (B) of Pt 58 were transfected with 100 nM siRNA oligonucleotides. Total RNA was extracted 4 days after transfection and qRT-PCR was performed using GAPDH as reference. The percentage of mRNA levels in siRNA treated samples vs. control siRNA treated samples was calculated as: 2 (CT(Control si - GAPDH) - CT(Target si - GAPDH))×100%. (TIF) [file pone.0046685.s007.tif]

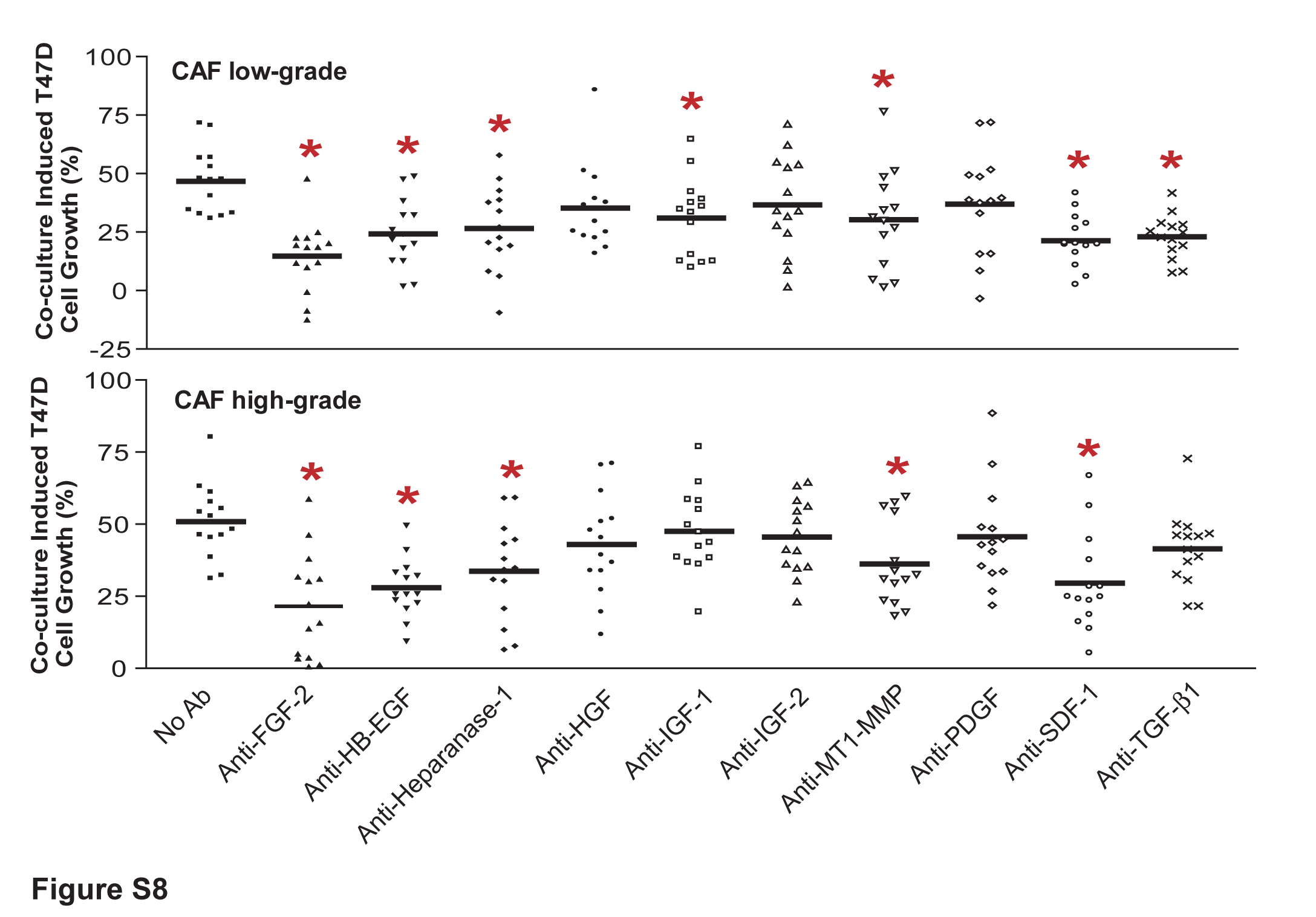

Supplement: Figure S8 — Scatter plot representation of the data shown in Figure 4A . (TIF) [file pone.0046685.s008.tif]

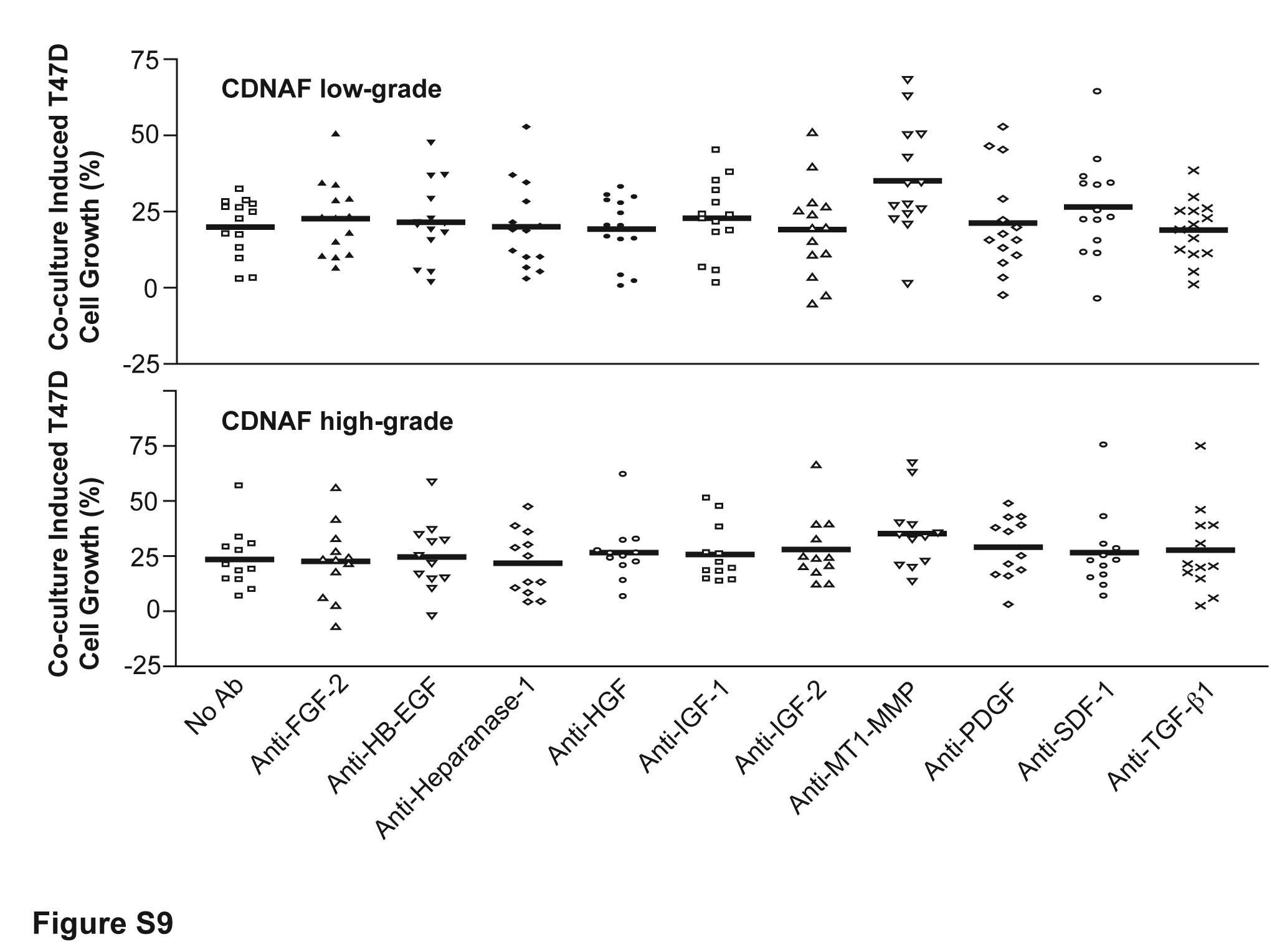

Supplement: Figure S9 — Scatter plot representation of the data shown in Figure 5A . (TIF) [file pone.0046685.s009.tif]
