# Supplementary material for: Functional Screen of Paracrine Signals in Breast Carcinoma Fibroblasts
Source: PLoS One. 2012 Oct 8;7(10):e46685. doi: 10.1371/journal.pone.0046685 (PMC3466317; doi:10.1371/journal.pone.0046685)
Supplement: Table S1 — Panel of neutralizing antibodies used in the co-culture screen. (DOC) [file pone.0046685.s010.doc]

Table S1: Panel of neutralizing antibodies used in the co-culture screen.

| Target | Source Species | Company | Cat # | Working Conc. (μg/ml) |
| --- | --- | --- | --- | --- |
| FGF-2 | Goat | R&D | AF-233-NA | 10 |
| HB-EGF | Goat | R&D | AF-259-NA | 10 |
| Heparanase 1 | Rabbit | Abcam | Ab42871 | 10 |
| HGF | Goat | R&D | AF-294-NA | 10 |
| IGF-1 | Goat | R&D | AF-291-NA | 25 |
| IGF-2 | Goat | R&D | AF-292-NA | 25 |
| MT1-MMP | Rabbit | Abcam | Ab38970 | 10 |
| PDGF | Rabbit | R&D | AB-20-NA | 100 |
| SDF-1 | Goat | R&D | AF-310-NA | 10 |
| TGF-β1 | Goat | R&D | AF-246-NA | 10 |
| Wnt1 | Rabbit | Abcam | Ab63934 | 10 |
