# Supplementary material for: Functional Screen of Paracrine Signals in Breast Carcinoma Fibroblasts
Source: PLoS One. 2012 Oct 8;7(10):e46685. doi: 10.1371/journal.pone.0046685 (PMC3466317; doi:10.1371/journal.pone.0046685)
Supplement: Table S2 — Characteristics of carcinomas as source of CAF and NF. (DOC) [file pone.0046685.s011.doc]

Table S2: Characteristics of carcinomas as source of CAF and NF.

|  | | ***Number of Cases*** | ***% of Total Cases*** |
| --- | --- | --- | --- |
| **Tumor Subtype** | Ductal | 23 | 82% |
|  | Lobular | 5 | 18% |
| **Tumor Grade** | I | 6 | 21% |
|  | II | 8 | 29% |
|  | III | 14 | 50% |
| **Estrogen Receptor** | Positive | 21 | 75% |
|  | Negative | 6 | 21% |
|  | n/a | 1 | 4% |
| **Progesterone Receptor** | Positive | 18 | 64% |
|  | Negative | 8 | 29% |
|  | n/a | 2 | 7% |
| **ErbB-2**  **(Her-2)** | Positive | 5 | 18% |
|  | Negative | 22 | 78% |
|  | n/a | 1 | 4% |
