# Supplementary material for: Functional Screen of Paracrine Signals in Breast Carcinoma Fibroblasts
Source: PLoS One. 2012 Oct 8;7(10):e46685. doi: 10.1371/journal.pone.0046685 (PMC3466317; doi:10.1371/journal.pone.0046685)
Supplement: Table S3 — Relationship between pathologic grade or steroid hormone receptor status and T47D cell growth response to inhibiting IGF-1, TGF-â1 or HB-EGF. (DOC) [file pone.0046685.s012.doc]

Table S3: Relationship between pathologic grade or steroid hormone receptor status and T47D cell growth response to inhibiting IGF-1**,** TGF-β1 or HB-EGF.

| **Response to anti-IGF-1 in co-culture** | **Negative**  **(No Inhibition)** | **Positive**  **(Significant Inhibition)** | **Total** |
| --- | --- | --- | --- |
| **Grade 1 & 2** | 7 (50%) | 7 (50%) | 14 |
| **Grade 3** | 14 (100%) | 0 (0%) | 14 |
| **Total** | 21 | 7 | 28 |
| ***P* = 0.0058** |  |  |  |

| **Response to anti-TGF-β1 in co-culture** | **Negative**  **(No Inhibition)** | **Positive**  **(Significant Inhibition)** | **Total** |
| --- | --- | --- | --- |
| **Grade 1 & 2** | 5(36%) | 9 (64%) | 14 |
| **Grade 3** | 14 (100%) | 0 (0%) | 14 |
| **Total** | 19 | 9 | 28 |
| ***P* = 0.0006** |  |  |  |

| **Response to anti-HB-EGF in co-culture** | **Negative**  **(No Inhibition)** | **Positive**  **(Significant Inhibition)** | **Total** |
| --- | --- | --- | --- |
| **ER^-^ or PR^-^** | 0 (0%) | 8 (100%) | 8 |
| **ER^+^ and PR^+^** | 10 (56%) | 8 (44%) | 18 |
| **Total** | 10 | 16 | 26 |
| ***P* = 0.0095** |  |  |  |
